# Greater Compassion in Responding to Medical Error

**DOI:** 10.21315/mjms-03-2025-152

**Published:** 2025-04-30

**Authors:** Yusrita Zolkefli

**Affiliations:** PAPRSB Institute of Health Sciences, Universiti Brunei Darussalam, Brunei Darussalam

Dear Editor,

I carefully read the article by Yusof and Razali (1), who advocates for a transition from a culture of blame to a culture of learning in the context of medical error. The authors also highlighted the importance of acknowledging that clinical errors can happen at any time, even to the most knowledgeable and experienced healthcare professionals (1). In this commentary, I hope to reflect on some of the authors’ invaluable observations.

To begin with, the authors were correct in their assertion that it was essential to end the culture of blame (1). A working environment that prioritises punishment would only lead to further harm, for example, caused by hidden or unreported medical errors. Such a lack of transparency may happen because healthcare professionals do not feel comfortable openly reporting errors and near misses because of fear of retribution. Because of this, incidents are not documented in a timely or adequate manner (2). On the other hand, all of this would only serve to perpetuate a cycle of error, which would inevitably compromise patient care. I accept that there are healthcare professionals who have committed preposterous errors, therefore requiring the adoption of stringent measures. However, there is also an impetus for healthcare professionals to be in a safer environment that prioritises a trusting and just culture in which adverse events are acknowledged as valuable opportunities to explore contributing factors and learn lessons rather than being given immediate “guilty as charged” cards (3).

Meanwhile, the authors also encourage the provision of tangible support to the second victims (1), particularly considering the emotional and moral impacts that errors can have on the affected healthcare professionals. Psychological safety in healthcare professionals, for example, can be achieved when the team prioritises patient safety (4). Additionally, organisations have a salient duty to engage with healthcare professionals in their psychological recovery following a medical error. As an example, the consideration of TRUST, which is an acronym for the “Five Rights of the Second Victim,” is a critical strategy. Such strategy refers to the fundamental principles of support and treatment that should be given to healthcare professionals who have experienced a significant adverse patient event. Such rights include the following: Treatment That Is Just, Respect, Understanding, Support, and Transparency (5).

While there is currently an incremental effort to assist second victims, it is also vital to keep in mind that individual healthcare professionals are still accountable for their practice, as the authors have accurately asserted (1). Being identified as a “second victim” within the second-victim phenomenon does not detract them from adhering to a strong set of ethical values and standards. Among these obligations includes the duty to report colleagues who are deemed incompetent or have the potential to harm patients through medical error, to be honest when things go wrong, to report mistakes as incidents, and to seek help when mistakes occur. It also serves as a basis for greater accountability from organisations to ensure that the response to medical errors is driven by compassion, with the primary goal of learning from the error and preventing future errors.

In summary, Yusof and Razali (1) reinforced the urgency of abandoning the blame culture, which is commonly reported to be unhelpful for sustainable error management. That being said, the unsettling reality is that most healthcare professionals, if not all, would make unintentional human errors at any time in their practice. Therefore, to help the second victims achieve a meaningful recovery and flourish in their professional life, the healthcare organisation must address and manage this error with a greater sense of compassion and accountability.
